# Cross antigenicity of immunodominant polypeptides of somatic antigen of *Oesophagostomum columbianum* with other helminths by western blotting

**DOI:** 10.14202/vetworld.2015.1279-1285

**Published:** 2015-11-05

**Authors:** Sunita Dalal, Arvind Prasad, Abdul Nasir, Vijesh Kumar Saini

**Affiliations:** Network Program on G.I. Parasitism, Division of Parasitology, Indian Veterinary Research Institute, Izatnagar, Bareilly, Uttar Pradesh, India

**Keywords:** cross antigenicity, hyperimmune sera, immunodiagnostic polypeptides, *Oesophagostomum columbianum*

## Abstract

**Aim:**

*Oesophagostomum columbianum* in small ruminants in India is found as mixed infection commonly in sheep and goat. *Haemonchus contortus*, an abomasal nematode is found as concurrent infection with it. Eggs of *Haemonchus* and *O. columbianum* cannot be easily distinguished. Diagnosis of *O. columbianum* may only be possible if a non-cross antigenic polypeptide was available for immunodiagnosis.

**Materials and Methods:**

Somatic antigen (SoAg) of *O. columbianum* was fractionated by sodium dodecyl sulfate-polyacrylamide gel electrophoresis and immunodominant polypeptides were identified by western blotting with homologous hyperimmune serum (HIS) and experimental sera of sheep or goat infected with other helminths.

**Results:**

SoAg of *O. columbianum* was immunoaffinity purified. Sharp polypeptide bands of 130, 72 and 68 KDa were observed along with several faint bands of lower molecular weight. Western blot of purified SoAg of *O. columbianum* with homologous HIS showed reaction with all the protein bands of 17, 28, 30, 32, 35, 38, 50, 68, 100, 130, 150, and 170 kDa. For identification of non-cross antigenic polypeptide, immunoaffinity purified SoAg of *O. columbianum* was reacted to heterologous HIS against *H. contortus, Paramphistomum epiclitum*, and *Fasciola gigantica* in western blotting utilizing completely dry method (i-blot). Among high molecular weight polypeptides 100 and 150 kDa were non-cross antigenic and among low molecular weight except 50 kDa polypeptide, 17, 30, 32, 35, and 38 kDa of *O. columbianum* were not cross antigenic with other helminths.

**Conclusions:**

Hence, polypeptides of 17, 30, 32, 35 and 38 kDa as well as 100 and 150 kDa polypeptides of *O. columbianum* may be exploited for immunodiagnosis of the infection in sheep and goat with extensive studies on cross antigenicity.

## Introduction

*Oesophagostomum columbianum*, commonly known as pimply gut worm causes severe damage to the gut of sheep and goat causing diarrhea. It is one of the most prevalent highly pathogenic and economically important nematode parasite throughout the Asian subcontinent [1]. *O. columbianum* is more common in tropical and subtropical countries where temperature and humidity are much higher compared to temperate countries. It is also different from other nematodes in the respect that adults are not pathogenic residing in the lumen of the large intestine whereas L_4_ larvae remain in nodules developing around these [2] and pathogenicity is mainly due to these larvae. Prepatent period of *O. columbianum* is 41 days [3] which is longer than other gastrointestinal (GI) nematodes which in most of the cases is 2-3 weeks.

*O. columbianum* is found as mixed infection with other nematodes particularly *Haemonchus contortus* and other helminths like *Paramphistomum epiclitum* which also shares intestinal habitat with it. *Fasciola gigantica* is also found in some regions in India. Clinical symptoms of these infections are overlapping making specific diagnosis difficult. Although, studies on immunodiagnosis of *O. columbianum* was reported previously by few worker employing ELISA and double immunodiffusion (DID) test [4-8]. Antigenic cross-reactivity of *O*. *columbianum* with. *H. contortus* was reported using DID and similar results employing ELISA [4]. Surface reactivity patterns among strongylid nematodes using immunoperoxidase assay revealed cross antigenicity of *O. columbianum* with *H*. *contortus* and *Bunostomum trigonocephalum* [9].

Hence, immunodiagnosis with a suitable non-cross antigenic molecule may be a suitable alternative for specific diagnosis and timely treatment to prevent morbidity and mortality of infected animals. Cross antigenicity among mixed helminths infection has already been reported by Philipp and Rumjaneck [10]; Cuquerella *et al*. [11]; Molina *et al*. [12]. Keeping in view the pathogenic importance of *O. columbianum* in sheep and goat, the present study was conducted to identify non-cross antigenic polypeptide to be utilized for immunodiagnosis further.

## Materials and Methods

### Ethical approval

All the procedures have been carried out in accordance with the guidelines laid down by the Institutional/Animal Ethics Committee and in accordance with local laws and regulations.

### Collection of parasites

**Figure-1:**
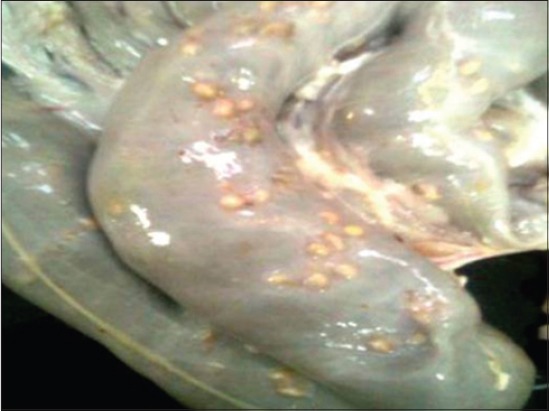
Pimply gut in colon of sheep.

Adult *O. columbianum* were collected from the intestines of infected sheep and goat having pimply gut (Figure-1) procured from the local abattoir at Bareilly (Uttar Pradesh) India. The parasites were washed several times in phosphate buffered saline (PBS - pH 7.2).

### Identification of *O. columbianum*

*O. columbianum* was identified as per their characteristic features [13,14].

### Preparation of somatic antigen (SoAg)

The adult worms were thoroughly homogenized using mortar and pestle in sufficient quantity of PBS (pH7.2), then sonicated and further centrifuged at 12000 rpm at 4°C for 25 min. The supernatant was collected and filtered through 0.2 μm (Sartorius) filter membrane. The filtrate was stored at –70°C with PMSF.

### Protein estimation

The protein concentration of the antigens was estimated as per Lowry’s method [15].

### Characterization of SoAg by sodium dodecyl sulfate-polyacrylamide gel electrophoresis (SDS-PAGE)

SDS-PAGE was performed to observe the polypeptide profile of the antigen of *O. columbianum*. Molecular weights of the polypeptides were determined with help of standard marker 11-170 KDa (Fermentas) as per the given method [16]. 40 µg of protein of each antigen sample was mixed with ×1 sample buffer solution in the ratio of 1:1. The antigens with sample buffer (50 µl) were charged into the wells of the gel. Molecular weight marker (10 µl) was also loaded in the respective well. After the electrophoretic run, the gel was carefully removed from the glass plate and stained with coomassie blue R-250 for 30 min until stained properly. It was followed by destaining until the polypeptide bands were clearly visible.

### Hyperimmune sera (HIS)

HIS was raised against SoAg of *O. columbianum* in PBS (pH 7.2) in New Zealand white rabbits as per the procedure with control [17]. Blood samples were collected from all the rabbits intracardially before immunization to obtain preimmunized normal rabbit sera and stored at –20°C. For raising HIS, stable water in oil emulsion of an equal volume of antigen Freuend’s complete and Freuend’s incomplete adjuvants were used as inoculums. Dose and route of administration of antigen and adjuvant were used as per standard protocol. Primary immunization (0 day) was done with subcutaneous injection with 450 µg/animal. Further, booster doses were given at an interval of 12 days with increasing dose of 50 µg protein from first to nine boosters by intramuscular injection. Finally, HIS were collected from immunized animals.

### Western blotting

Western blotting was performed using 7 min i-blot (Invitrogen). SDS-PAGE gel was removed carefully from the glass plates and kept in triple distilled water for 10 min. Polypeptides from the gel were transferred to nitrocellulose (NC) membrane (0.2 µm). The membrane was kept in blocking buffer (PBS-M) for 2 h and washed with washing solution (PBS-T) 3 times with 5 min interval. The membrane was incubated with anti - serum (1:100) at 37°C for 2 h and washed 3 times with washing solution. Secondary antibody with 1:10000 dilution (Goat anti- Rabbit IgG HRP conjugate) was added to the membrane and incubated at room temperature for 1 h followed by final washing. 3, 3’-diaminobenzidine substrate was further added for developing bands of reacting polypeptides.

### Immunoaffinity purification of SoAg

**Graph-1:**
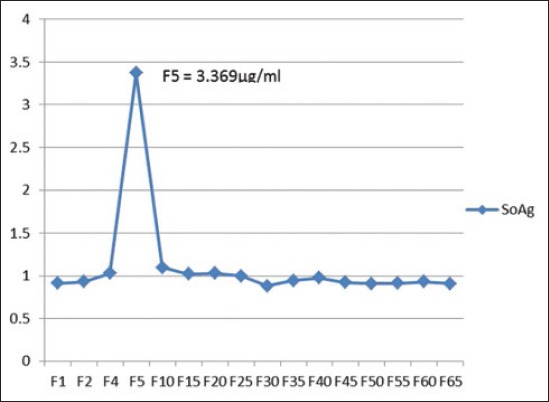
Elution pattern of bound fractions of purified somatic antigen of *Oesophagostomum columbianum*.

SoAg was purified using aminolink matrix (Pierce). The column containing matrix was equilibrated at room temperature and regenerated by washing with coupling buffer. The column was loaded with purified immunoglobulin (Ig) (5 mg/ml). IgG was separated from the HIS raised in rabbit against SoAg of *O. columbianum*. For purification of IgG, protein G based IgG purification kit (Merck) was used and from 10 ml HIS approximately 20 mg IgG was obtained. 20 mg purified Ig was loaded in the column and 200 μl of aminolink reductant solution was added. It was incubated overnight at 4°C with end to end shaking. Next day, the column was washed to remove excess uncoupled antibody. After complete washing, the antigen (5 mg/ml) was loaded to the column and incubated overnight at 4°C with end to end shaking. Excess uncoupled Ag was removed with washing buffer and finally the bound polypeptides were eluted utilizing elution buffer and fractions were collected. OD values of the fractions (Graph-1) were recorded, and the fractions of interest were pooled and concentrated followed by recording the OD values of pooled and concentrated fraction.

### SDS-PAGE of purified fractions

SDS-PAGE of immunoaffinity purified fractions of the SoAg was performed as described above.

### Western blotting for cross antigenicity

Western blot was performed with eluted purified fractions of SoAg with heterologous HIS as well as experimental sera (sheep and goat) raised against *H. contortus, P. epiclitum*, and *F. gigantica* and polypeptides showing reactivity to heterologous sera were identified for further identification of non-cross antigenic polypeptides in *O. columbianum* antigen. Experimental sera of sheep and goat against *H. contortus, P. epiclitum*, and *F. gigantica* were also utilized for western blotting.

## Results

### Identification of parasite

**Figure-2:**
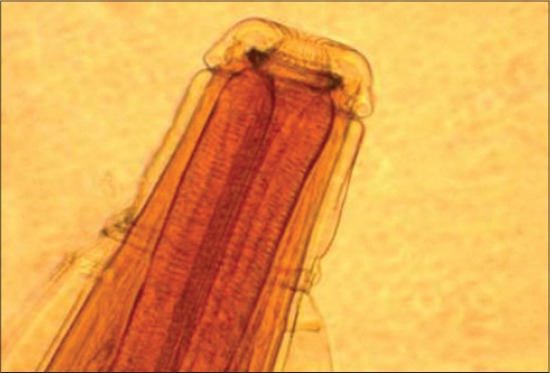
Anterior end of *Oesophagostomum columbianum* (cervical papillae and leaf crown).

The worms showed cuticle forming mouth collar in form of truncated cone. The cervical groove was extending around ventral surface of the body and cuticle anterior to this groove inflated to form cephalic vesicle. Lateral alae originated immediately behind cervical groove, extending almost whole length of the body and cervical papillae pierced the anterior extremities of the lateral alae. The buccal capsule was shallow, external corona radiata consisting of 20-24 elements with internal corona radiata having two small elements to each element of external leaf crown. Oesophagus was club-shaped with oesophageal intestinal valve. Male bursa was well-developed with equal sized spicules not extending from bursal lobe. Vulva of a female was slightly prominent opening little anterior to anus. Vagina was very short, transverse, leading into kidney shaped ovijectors or pars ejectrix. The morphological features confirmed to the description of *O. columbianum* (Figure-2).

### Antigen preparation and protein concentration

SoAg was prepared by standard protocol and the protein concentration was 3.32 mg/ml as per Lowry method.

### Antigen characterization of O. columbianum by SDS-PAGE

Protein profile of SoAg of adult *O. columbianum* as determined by 12% SDS-PAGE revealed prominent protein bands of 11-130 kDa.

### Identification of immunodominant polypeptides in SoAg of *O. columbianum* by western blotting

Protein bands fractionated by SDS-PAGE were subjected to western blotting using HIS raised against So Ag. Polypeptide bands of 17, 28, 30, 32, 35, 38, 50, 68, 100, 130, and 150 KDa showed strong reactivity and were designated as immunodominant polypeptides.

### SDS-PAGE of immunoaffinity purified SoAg

**Figure-3:**
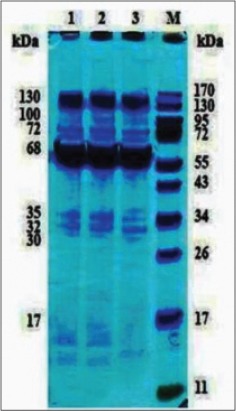
Sodium dodecyl sulfate-polyacrylamide gel electrophoresis of immunoaffinity purified somatic antigen of *Oesophagostomum columbianum*, Lane: 1, 2 and 3: Immuno affinity purified fraction, Lane: M: Moecular weight marker.

Protein profile of purified fractions of SoAg was determined by 12% SDS-PAGE. Sharp bands of 130, 72 and 68 kDa could be observed along with several faint bands of lower molecular weight (Figure-3).

### Western blot with immunoaffinity purified SoAg of *O. columbianum* with homologous HIS

**Figure-4:**
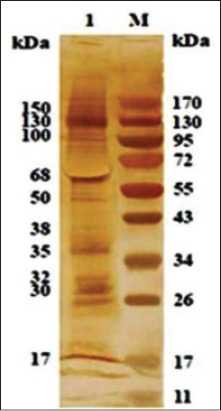
Western blotting of immunoaffinity purified somatic antigen of *Oesophagostomum columbianum* with homologous hyperimmune serum.

Western blot of purified SoAg of *O. columbianum* with homologous HIS showed reaction with all the protein bands of 17, 28, 30, 32, 35, 38, 50, 68, 100, 130, 150 and 170 kDa (Figure-4).

### Immunoaffinity purified SoAg of *O. columbianum* and HIS against *H. contortus* SoAg

**Figure-5:**
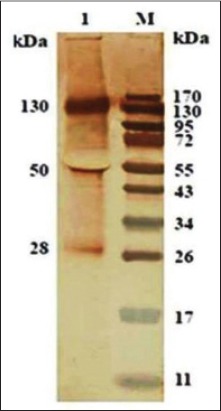
Cross-antigenicity of immunoaffinity purified somatic antigen of *Oesophagostomum columbianum* with *anti-Haemonchus contortus* antibody (hyperimmune serum) in western blotting.

HIS against *H. contortus* reacted to 130, 50, and 28 KDa polypeptide present in the immunoaffinity purified SoAg of *O*. *columbianum* (Figure-5).

### Immunoaffinity purified SoAg of *O. columbianum* and experimental sera of sheep against *H. contortus*

**Figure-6:**
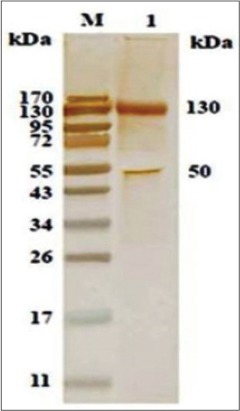
Cross antigenicity of immunoaffinity purified somatic antigen of *Oesophagostomum columbianum* with experimental serum of sheep infected with *Haemonchus contortus* in western blotting.

The experimental serum of sheep against *H. contortus* reacted only to 130 and 50 KDa polypeptide of purified SoAg of *O. columbianum* (Figure-6).

### Immunoaffinity purified SoAg of *O. columbianum* and HIS against *P. epiclitum*

**Figure-7:**
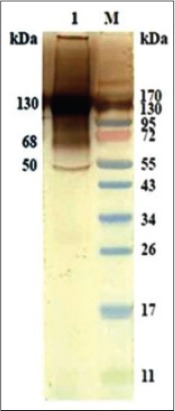
Cross antigenicity of immunoaffinity purified somatic antigen of *Oesophagostomum columbianum* with anti-*Paramphistomum epiclitum* antibody (hyperimmune serum) in western blotting.

HIS against *P. epiclitum* reacted to polypeptides of 130, 68 and 50 KDa of purified SoAg of *O. columbianum* (Figure-7).

### Immunoaffinity purified SoAg of *O. columbianum* and experimental sera of goat against *P. epiclitum*

**Figure-8:**
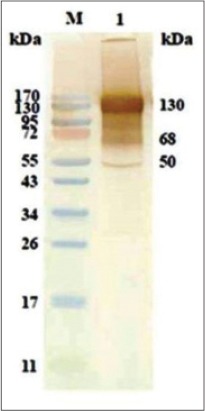
Cross-antigenicity of immunoaffinity purified somatic antigen of *Oesophagostomum columbianum* with experimental serum of *Paramphistomum epiclitum* in western blotting.

The experimental serum of goat against *P. epiclitum* reacted to 130, 68, and 50 KDa polypeptides in the purified SoAg of *O. columbianum* (Figure-8).

### Immunoaffinity purified SoAg of *O. columbianum* and experimental sera of sheep against *F. gigantica*

**Figure-9:**
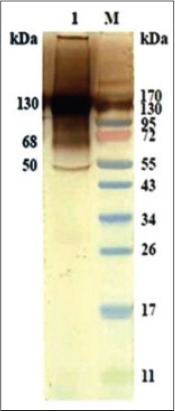
Cross-antigenicity immunoaffinity purified somatic antigen of *Oesophagostomum columbianum* with experimental serum of Fasciola gigantica in western blotting.

Experimental serum of sheep against *F. gigantica* reacted with 130, 68 and 50 KDa polypeptides of purified SoAg of *O. columbianum* (Figure-9).

The results of cross antigenicity of *O. columbianum* have been summarized in Table-1.

**Table-1:** Cross-antigenicity of different helminthes with immunoaffinity purified SoAg of *O. columbianum*.

Heterologous serum	Cross antigenic polypeptide (kDa)
*P. epiclitum* (HIS)	130, 68, 50
*P. epiclitum* (experimental serum in goat)	130, 68, 50
*H. contortus* (HIS)	130, 50, 28
*H. contortus* (experimental serum in sheep)	130, 50
*F. gigantica* (experimental serum in goat)	130, 68, 50

*P. epiclitum=Paramphistomum epiclitum, F. gigantica=Fasciola gigantica, H. contortus=Haemonchus contortus, O. columbianum=Oesophagostomum columbianum*, SoAg=Somatic Antigen, HIS=Hyperimmune serum

## Discussion

Non-cross antigenic immunodominant polypeptides in SoAg of *O. columbianum* were identified by western blotting. In affinity purified fraction, major prominent protein bands were 68, 72, 100, and 130 kDa, whereas low molecular weight polypeptides formed feeble bands. Semi dry method for western blotting is routinely used to transfer polypeptides from SDS-PAGE gel, but incomplete transfer of polypeptides was frequently encountered. To overcome the problem, completely dry method i-blot (Invitrogen) was utilized. This method had several advantages and most important was time interval (7 min) as compared to semi-dry method taking few hours for complete transfer of polypeptides. Therefore, for cross antigenicity studies completely dry method was utilized with the objective that all the polypeptides transferred were available for reaction to antibodies. It was observed that in western blotting polypeptides of lower as well as higher molecular weight were transferred to NC membrane and all the antigenic polypeptides on the membrane reacted to antibodies. During this study, the polypeptides of higher molecular weight (68, 72, 100, 130, and 150 kDa) and lower molecular weight (17, 30, 32, 35, and 38 kDa) were observed in the SDS-PAGE of affinity purified fraction of *O. columbianum*. During the study, it was found that polypeptides of lower molecular weight (17, 30, 32, 35, and 38 kDa) reacted to HIS against *O. columbianum* (homologous sera) but not to heterologous sera in western blotting. Besides, few polypeptides of higher molecular weight (100 and 150 kDa) also did not react to heterologous sera in western blotting raised against *H. contortus, P. epiclitum* and *F. gigantica*. It was inferred that there were several polypeptides both of lower as well as higher molecular weight in the SoAg of *O. columbianum* which were not shared with other helminths. In a similar study [18] found that out of 46 goats, five goats, which were negative for *O. columbianum*, showed false positive reaction in the assay since the goats although negative for *O. columbianum* had heavy worm burden of *H. contortus* (170-265) and *Trichuris ovis* (143-239). The authors suspected possibility of cross antigenicity with *H. contortus* and *T. ovis* due to reaction between HIS against ESAg-Oc and coproantigen (cAg) of *H. contortus* and *T. ovis*. The observation of these workers was found true during the present study because in western blotting, 50 kDa polypeptide of *O. columbianum* reacted with the HIS and experimental heterologous sera against *H. contortus, P*. *epiclitum*, and *F*. *gigantica* which confirmed cross antigenicity of *O. columbianum* with these helminths. Jas *et al*. [18] did not perform cross antigenicity studies. 39 and 50 kDa polypeptides were found immunodominant in the ES product of *O. columbianum* in western blotting with homologous HIS [18] however, 29, 38 and 49 kDa were found immunodominant by Arunkumar [19]. During our study, it was found that 50 kDa polypeptide of SoAg of *O. columbianum* was cross antigenic with *H. contortus, P. epiclitum*, and *F. gigantica*. This was actually the reason that goats negative for *O. columbianum* were giving false positive reaction in ELISA [18].

During this study, it was found that cross antigenicity of purified fraction of *O. columbianum* with *H. contortus*, *P. epiclitum*, and *F. gigantica* were mainly among high molecular weight polypeptides. Results of western blotting with heterologous hyperimmune sera and experimental sera of sheep and goat revealed that not only polypeptides of low molecular weight (17, 30, 32, 35, and 38 kDa) but also of high molecular weight (100 and 150 kDa) were non-cross antigenic to other helminths.

Sharing of polypeptides among GI nematodes has already been described by Siefker and Rickard [20], who recorded sharing of carbohydrate epitopes in an intestinal protein of bovine GI nematodes. In western blotting, these workers recognized sharing of high molecular weight protein bands ranging between 111 -298 KDa of *Haemonchus placei* of cattle with several nematodes such as *Ostertagia ostertagi*, *Cooperia punctata, H. contortus*, and *Oesopohagostomum radiatum*. These polypeptides were conserved in these species. In *O. radiatum* the polypeptides were semi-conserved. They also confirmed their finding with immunohistochemical studies verifying the intestinal location of the epitopes of the antigens. Cross antigenicity of *O. columbianum* during the present study was found with common trematodes *F. gigantica* and *P. epiclitum* in western blotting. Cross antigenicity of SoAg of *H*. *contortus* with *O. columbianum* and *F. gigantica* has already been reported by Prasad *et al*. [21] but not with *P. epiclitum*. Non-cross antigenic polypeptides in the SoAg were mostly low molecular weight polypeptides. Results indicate that for immunodiagnosis of *O. columbianum* infection, low molecular weight polypeptides 17, 30, 32, 35, and 38 kDa may be exploited. Jas *et al*. [18] found only 39 kDa polypeptide of ES antigen reacting to homologous hyperimmune sera against *O.columbianum*. Other polypeptides like 32 and 35 kDa probably were not available for recognition by antibodies due to failure of transfer during western blotting. However, during the present study, high molecular weight polypeptides of 100 and 150 kDa were also found non-cross antigenic.

## Conclusion

Immunodiagnosis of *O.columbianum* is rather difficult proposition since not much work has been done due to its importance mainly in tropical and subtropical countries The infection causes pimply gut in sheep and goat and causes severe diarrhea. Low molecular weight polypeptides of 17, 30, 32, 35, and 38 kDa were found non-cross antigenic with *H. contortus, P. epiclitum, and F. gigantica* in western blotting utilizing heterologous HIS, as well as experimental sera. Among high molecular weight polypeptides, 100 and 150 kDa were found non-cross antigenic. Further, work is therefore needed to explore diagnostic potential of these polypeptides with extensive studies on cross antigenicity to develop a specific immunodiagnostic test for *O. columbianum* infection in sheep and goat.

## Authors’ Contributions

SD: Preparation of antigen, SDS PAGE, purification of antigen and cross antigenicity studies. AP: Western blotting, cross antigenicity studies and preparation of manuscript. AN: Immunoaffinity purification raising of hyperimmune serum in rabbit and preparation of manuscript. VKS: Preparation of manuscript and data analysis.
